# A case of coexisting heterozygous *NOTCH3* and *HTRA1* mutations in cerebral small vessel disease

**DOI:** 10.1038/s41439-025-00317-z

**Published:** 2025-07-09

**Authors:** Masataka Yamashiro, Daigo Yasutomi, Yuichiro Ohya, Satoshi Ohyama, Hiroshi Takashima, Takashi Tokashiki

**Affiliations:** 1 Department of Neurology, National Hospital Organization Okinawa Hospital, Okinawa, Japan; 2 Department of Neurology and Geriatrics, Kagoshima University Graduate School of Medical and Dental Sciences, Kagoshima, Japan

**Keywords:** Genetics of the nervous system, Disease genetics

## Abstract

Hereditary cerebral small vessel diseases (CSVDs) include cerebral autosomal dominant arteriopathy with subcortical infarcts and leukoencephalopathy (CADASIL) caused by *NOTCH3*, cerebral autosomal recessive arteriopathy with subcortical infarcts and leukoencephalopathy caused by biallelic *HTRA1*, and heterozygous *HTRA1*-related CSVD. Here we report a case of a 53-year-old Japanese woman with coexisting *NOTCH3* p.R75P and *HTRA1* p.R166L mutations, each in the heterozygote. She presented with early-onset spastic paraparesis, frequent urination, cognitive impairment and baldness. We compared the clinical features of this case with known phenotypes of CADASIL caused by p.R75P, *HTRA1*-related CSVD. We reported cases with heterozygous *HTRA1* p.R166L to discuss the potential synergistic effects of the coexisting variants.

Cerebral autosomal dominant arteriopathy with subcortical infarcts and leukoencephalopathy (CADASIL) is the most common hereditary cerebral small vessel disease (CSVD), caused by pathogenic variants in the *NOTCH3* gene^1^. It typically presents with ischemic attacks, migraine with aura, mood disturbances and cognitive decline^1^. Characteristic magnetic resonance imaging (MRI) findings include white matter hyperintensities in the temporal poles and external capsules^1^. Histopathologically, granular osmiophilic material (GOM) in small arteries is a hallmark feature^1^.

CSVD related to *HTRA1* mutations includes both cerebral autosomal recessive arteriopathy with subcortical infarcts and leukoencephalopathy (CARASIL), caused by biallelic *HTRA1* variants, and heterozygous *HTRA1*-related CSVD^2^. CARASIL is characterized by early-onset ischemic strokes, cognitive decline, alopecia and spondylosis deformans^2^. By contrast, heterozygous *HTRA1* variants typically result in a later-onset, milder phenotype, but still show overlapping features such as stroke and white matter changes^2^.

We herein report the case of a 53-year-old Japanese woman with coexisting *NOTCH3* p.R75P and *HTRA1* p.R166L mutations, each in the heterozygote.

A 53-year-old woman gradually developed a gait disturbance at the age of 43 years. At 48 years of age, she began to experience falls and dysarthria. At 51 years of age, she began to suffer from frequent urination. At 52 years of age, her gait disturbance worsened, and she had difficulty going out. Cervical spondylotic myelopathy was suspected, and a laminectomy was performed. However, her gait disturbance continued to worsen postoperatively, and she was referred to our hospital at 53 years of age.

Her medical history included only Graves’ disease, which was well controlled. She was a past smoker until the age of 35 years but had no other vascular risk factors such as hypertension, diabetes mellitus or dyslipidemia.

She was Japanese, and her parents were not consanguineous. Her father died of a stroke in his 40s, and her mother, who had received dialysis treatment for renal failure, died at the age of 80 years (Fig. 2a). She had four siblings, two of whom, including herself, had articulation disorders. She had not been good at either her studies or sports in childhood. After graduating from high school, she changed jobs frequently.

She exhibited noticeable baldness compared with individuals of the same age and sex (Fig. 1a); did not experience any back pain; and was underweight, with a body mass index of 19 kg/m^2^. Neurological examination revealed mild cognitive impairment, with scores of 28 on the Mini-Mental State Examination, 15 on the Frontal Assessment Battery and 21 on the Japanese version of the Montreal Cognitive Assessment. A cranial nerve examination revealed horizontal gaze-evoked nystagmus and slurred speech. In the motor system, pyramidal tract signs with clumsiness were observed in both her upper and lower limbs, predominantly on the right side. Although no muscle weakness was present, deep tendon reflexes were markedly hyperactive in all her limbs, with positive Babinski signs and clonus. Autonomic dysfunction manifested as frequent urination and constipation.

**Fig. 1:**
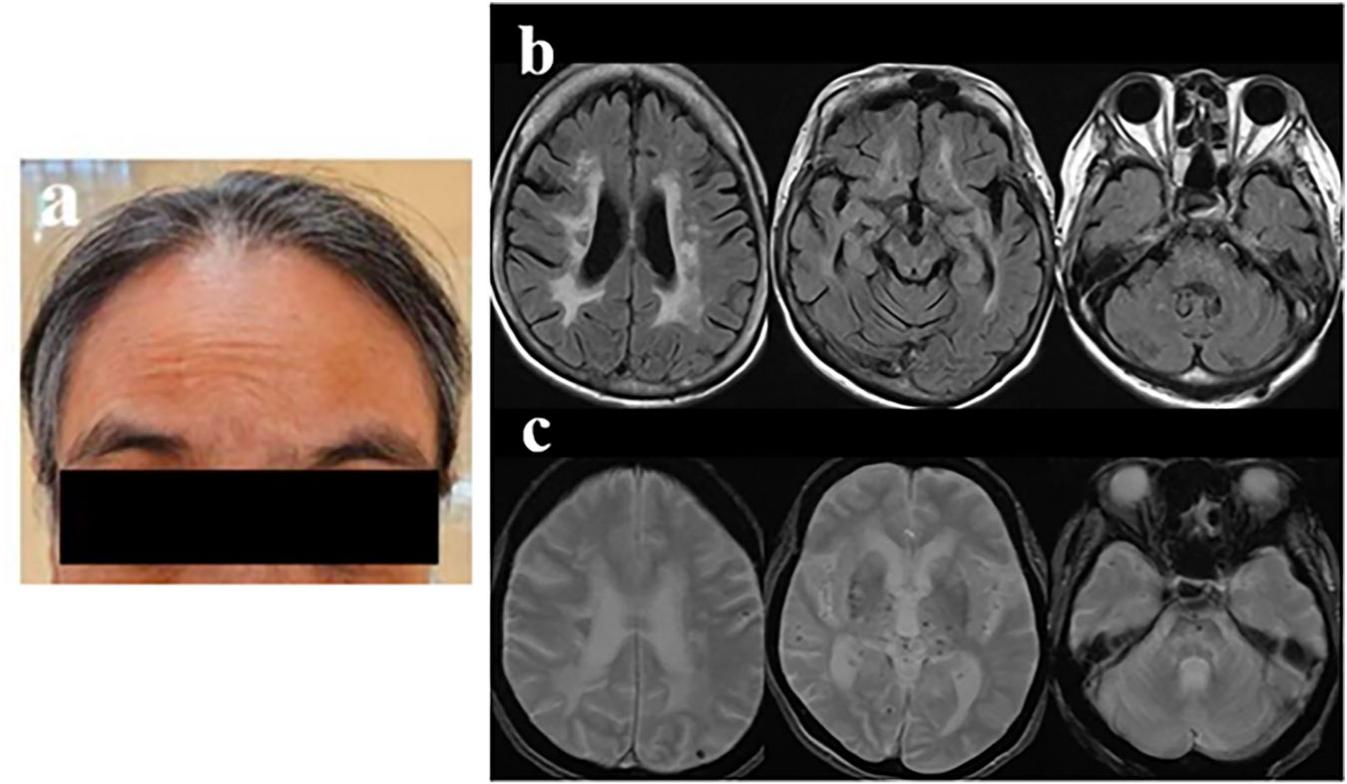
Clinical and neuroradiological features of the patient. **a** She exhibited noticeable baldness compared with individuals of the same age and sex. **b** Brain MRI showed scattered FLAIR hyperintense lesions in the cerebral cortex, corona radiata, basal ganglia, midbrain and pons. **c** Brain MRI revealed scattered microbleeds visualized as T2*-weighted hypointense lesions in the cerebral white matter, thalamus, midbrain and pons. A mild FLAIR hyperintensity was detected in the left temporal pole.

Blood tests revealed no renal dysfunction, and urine tests indicated no proteinuria. Cerebrospinal fluid analysis results were within normal limits. Electrocardiography and transthoracic echocardiography exhibited no risk of a cardiogenic embolism. Nerve conduction studies showed no evidence of neuropathy. Brain MRI revealed scattered fluid-attenuated inversion recovery (FLAIR) hyperintensities in the cerebral white matter, corona radiata, basal ganglia, midbrain and pons, and microbleeds (T2*-weighted hypointensities) in the white matter, thalamus, midbrain and pons. In addition, a mild FLAIR hyperintensity was detected in the left temporal pole (Fig. 1b, c). Vascular evaluation using contrast-enhanced computed tomography of the neck to the pelvis and magnetic resonance angiography of the brain revealed no arteriosclerotic stenosis of medium or large vessels. She had never experienced a symptomatic cerebral ischemic attack, and diffusion-weighted imaging on admission showed no evidence of acute cerebral infarction. Spinal MRI findings were unremarkable. The patient had a low risk of atherosclerosis, a family history of stroke, and early-onset and progressive CSVD; therefore, hereditary CSVD was suspected. Genetic analyses were performed using gene panel analysis and Sanger sequencing. The panel was designed to target genes associated with hereditary leukoencephalopathy and dementia. The identified variants were confirmed by both next-generation sequencing and Sanger sequencing.

**Fig. 2:**
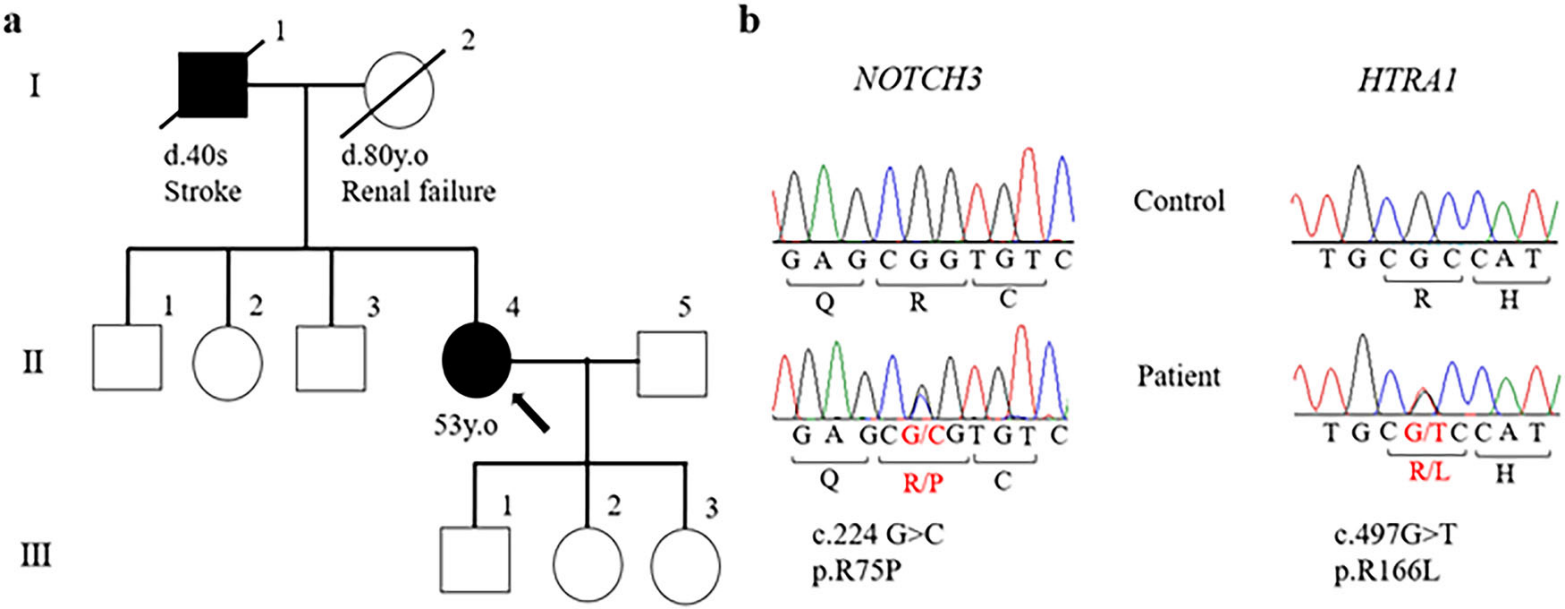
Pedigree of the studied family and genetic features of the patient. **a** Pedigree of the studied family. Solid symbols: affected individuals, open symbols: unaffected individuals. The proband (II-4) is indicated with an arrow. **b** Genetic testing confirmed these variants.

Clinical and molecular genetic studies were conducted with the approval of the Ethics Committee of the Graduate School of Medical and Dental Sciences at Kagoshima University. Informed consent was obtained from the patient’s daughters, and the genetic testing was explained in detail to the patient and her family. Genetic analysis identified mutations in *NOTCH3* (c.224G>C, p.R75P, heterozygous) and *HTRA1* (c.497G>T, p.R166L, heterozygous) (Fig. 2b).

This case involved CSVD with coexisting heterozygous mutations in *NOTCH3* and *HTRA1*, which had not been previously reported. In this case, the phenotype showed similarities and differences compared with reported phenotypes of *NOTCH3* p.R75P alone and *HTRA1* p.R166L alone. Similarities with heterozygous mutations include the presence of multiple thalamic hemorrhages on MRI, consistent with the known characteristics of the *NOTCH3* p.R75P mutation^3^. However, a mild temporal pole lesion was observed, which is less frequently seen in patients with the *NOTCH3* p.R75P mutation than in other *NOTCH3* mutations^3^. No studies report specific imaging features for heterozygous mutations in *HTRA1* p.R166L^4,5^.

By contrast, a notable difference compared with single heterozygous mutations was the younger age of onset observed in this case. Previous reports indicate that the mean age of onset for *NOTCH3* p.R75P and *HTRA1* p.R166L mutations is 55.1 and 58 years, respectively, with diagnosis occurring at 61.0 and 64 years^3–5^. This case exhibited an earlier onset (45 years) and diagnosis (55 years), approximately 10 years younger than the reported averages.

Frequencies of young-onset baldness in heterozygous *HTRA1* and CARASIL were 13.2% and 85.7%, respectively^2^. Of the three cases with heterozygous *HTRA1* p.P166L, one did not have baldness^4^, whereas there was no information in the other two cases^5^. These suggest that, in this case, young-onset baldness and disease onset younger than expected from reported phenotypes with each heterozygous mutation alone may be due to a synergic effect of the two mutations. The mechanism underlying the synergic effect in this case may be explained as follows. In *HTRA1*-related CSVD, a decrease in *HTRA1* protease activity leads to an accumulation of *HTRA1* substrates, resulting in elevated transforming growth factor (TGF)-β levels (a marker of vascular fibrosis) and the progression of vascular fibrosis^6^. In addition to the well-known mechanism of CADASIL involving the accumulation of the *NOTCH3* extracellular domain, which leads to the deposition of GOM in blood vessels, one recent study has reported that *HTRA1* substrates also accumulate in blood vessels in CADASIL^7^. In this case, the coexistence of *NOTCH3* and *HTRA1* gene mutations was hypothesized to further reduce *HTRA1* protease activity. The *HTRA1* p.R166L mutation is known to reduce enzymatic activity^8^, and the *NOTCH3* p.R75P mutation may contribute to the sequestration of *HTRA1* protein within GOM deposits^7^. This combined effect may lead to a more pronounced reduction in *HTRA1* activity than heterozygous *HTRA1*-related CSVD. As a result, the accumulation of *HTRA1* substrates may increase, potentially elevating TGF-β levels. As TGF-β is known to induce apoptosis in hair follicles^9^, this may be a plausible explanation for the baldness observed in this case.

A major limitation of this study is the lack of genotype information for *NOTCH3* p.R75P and *HTRA1* p.R116L in other family members. Further observations of this family are necessary to clarify the nature of this synergistic effect.

## HGV Database

The relevant data from this Data Report are hosted at the Human Genome Variation Database at 10.6084/m9.figshare.hgv.351210.6084/m9.figshare.hgv.3515.

## References

[1] Tikka, S. et al. CADASIL and CARASIL. *Brain Pathol.* **24**, 525–544 (2014). 25323668 10.1111/bpa.12181 PMC8029192

[2] Uemura, M. et al. HTRA1-related cerebral small vessel disease: review of the literature. *Front. Neurol.* **11**, 545 (2020). 32719647 10.3389/fneur.2020.00545 PMC7351529

[3] Takei, J. et al. Microbleed clustering in thalamus sign in CADASIL patients with NOTCH3 R75P mutation. *Front. Neurol.* **14**, 1241678 (2023). 37681004 10.3389/fneur.2023.1241678 PMC10480842

[4] Verdura, E. et al. Heterozygous HTRA1 mutations are associated with autosomal dominant cerebral small vessel disease. *Brain* **138**, 2347–2358 (2015). 26063658 10.1093/brain/awv155

[5] Cao, H. et al. A novel heterozygous HTRA1 mutation in an Asian family with CADASIL-like disease. *J. Clin. Lab Anal.* **36**, e24174 (2022). 34951056 10.1002/jcla.24174 PMC8841136

[6] Nozaki, H. Consideration of the pathogenesis of CARASIL. *Rinsho Shinkeigaku* **52**, 1360–1362 (2012). 23196618 10.5692/clinicalneurol.52.1360

[7] Zellner, A. et al. CADASIL brain vessels show a HTRA1 loss-of-function profile. *Acta Neuropathol.* **136**, 111–125 (2018). 29725820 10.1007/s00401-018-1853-8

[8] Uemura, M. et al. HTRA1 mutations identified in symptomatic carriers have the property of interfering the trimer-dependent activation cascade. *Front. Neurol.* **10**, 693 (2019). 31316458 10.3389/fneur.2019.00693 PMC6611441

[9] Shin, H. et al. Induction of transforming growth factor-beta 1 by androgen is mediated by reactive oxygen species in hair follicle dermal papilla cells. *BMB Rep.* **46**, 460–464 (2013). 24064061 10.5483/BMBRep.2013.46.9.228 PMC4133876

